# Numerical integration methods and layout improvements in the context of dynamic RNA visualization

**DOI:** 10.1186/s12859-017-1682-0

**Published:** 2017-05-30

**Authors:** Boris Shabash, Kay C. Wiese

**Affiliations:** School of Computing Science, Simon Fraser University, 8888 University Drive, Burnaby, BC Canada

**Keywords:** RNA, Visualization, Graph layout, Numerical integration

## Abstract

**Background:**

RNA visualization software tools have traditionally presented a static visualization of RNA molecules with limited ability for users to interact with the resulting image once it is complete. Only a few tools allowed for dynamic structures. One such tool is jViz.RNA. Currently, jViz.RNA employs a unique method for the creation of the RNA molecule layout by mapping the RNA nucleotides into vertexes in a graph, which we call the detailed graph, and then utilizes a Newtonian mechanics inspired system of forces to calculate a layout for the RNA molecule. The work presented here focuses on improvements to jViz.RNA that allow the drawing of RNA secondary structures according to common drawing conventions, as well as dramatic run-time performance improvements. This is done first by presenting an alternative method for mapping the RNA molecule into a graph, which we call the compressed graph, and then employing advanced numerical integration methods for the compressed graph representation.

**Results:**

Comparing the compressed graph and detailed graph implementations, we find that the compressed graph produces results more consistent with RNA drawing conventions. However, we also find that employing the compressed graph method requires a more sophisticated initial layout to produce visualizations that would require minimal user interference. Comparing the two numerical integration methods demonstrates the higher stability of the Backward Euler method, and its resulting ability to handle much larger time steps, a high priority feature for any software which entails user interaction.

**Conclusion:**

The work in this manuscript presents the preferred use of compressed graphs to detailed ones, as well as the advantages of employing the Backward Euler method over the Forward Euler method. These improvements produce more stable as well as visually aesthetic representations of the RNA secondary structures. The results presented demonstrate that both the compressed graph representation, as well as the Backward Euler integrator, greatly enhance the run-time performance and usability. The newest iteration of jViz.RNA is available at https://jviz.cs.sfu.ca/download/download.html.

**Electronic supplementary material:**

The online version of this article (doi:10.1186/s12859-017-1682-0) contains supplementary material, which is available to authorized users.

## Background

### RNA and its structure

Ribo-nucleic Acid (RNA) is a polymer of nitrogenous bases, composed mainly of Adenine, Cytosine, Guanine, and Uracil (denoted as A, C, G and U, respectively). RNA is very similar to Deoxyribo-nucleic Acid (DNA) in its basic composition, but while DNA is regularly found as two complimentary strands, RNA can be found as a single strand of nucleotides. This primary structure (the string of nucleotides) then folds over itself into a secondary structure when the bases in the RNA strands pair up via hydrogen bonding. The RNA molecule can then twist, fold, or otherwise change its conformation in 3D space, giving it a functional three dimensional form, known as the tertiary structure.

Single stranded, functional RNA is an important agent in many biological processes. From humans to bacteria and viruses, there are many examples of RNA molecules that are important to understand, classify, and research. Some notable examples include RNA motifs that allow viruses to manipulate host replication machinery [1–5], bacterial RNA motifs that give rise to antibiotic resistance [6, 7], and man-made RNA molecules designed for therapeutics [8].

### RNA secondary structure visualization

There are many RNA visualization tools that have been developed, and two excellent reviews of them can be found in [9, 10], with notable examples that are still available including VARNA [11], jViz.RNA [12–16], Forna [17], PseudoViewer [18–22], 4SALE [23, 24], Assemble2 [25, 26], RNA2DMap [27], R2R [28], and R-Chie [29]. All Visualization software developed for RNA have as their goal to display an informative structure of the RNA molecule, usually focusing on its secondary structure, that can be annotated and used to convey information in presentations, publications, and any other two-dimensional media.

However, the majority of RNA visualization software designed produce a static layout of the RNA molecule that may not be ideal for the user. While for small RNA molecules such as transfer RNA (tRNA), this problem almost never arises, for large RNA molecules, this layout can be such that sections of the RNA overlap each other, making annotation of certain regions problematic and uninformative. There are only three notable examples that create dynamic layouts which are responsive to user interactions: jViz.RNA, PseudoViewer, and VARNA.

The designers of VARNA do not explicitly state how they construct the RNA molecule and how the algorithm responsible for user response behaves, but one can estimate how the algorithm operates from interacting with the software as a web applet [30]. The RNA structure is translated into a graph where loops make up the vertexes and stems make up the edges. Thus, by dragging the stems, the user can arrange the layout of the RNA molecule. While this allows users to fully customize the RNA layout to their needs. The high degree of user involvement might make the task seem very daunting for large RNA molecules.

PseudoViewer is another application that allows its users to manipulate the RNA structure. Originally an application designed focusing on RNA pseudoknots, PseudoViewer puts a great deal of effort into creating the initial layout of the RNA molecule. Additionally, the user can manipulate the RNA structural motifs. However, certain manipulations of the RNA conformation destabilize the system and cause the RNA model to break apart. Furthermore, since PseudoViewer’s focus is primarily on pseudoknotted RNA structures, it tends to arrange the RNA structure layout focusing very heavily on clear display of pseudoknot types, at the expense of the aesthetics related to the rest of the structure.

jViz.RNA, a third software designed for dynamic RNA visualization, employs a different approach to creating the RNA model. jViz.RNA translates the RNA molecule into a graph as well, but maps each nucleotide to a vertex and each bond between nucleotides (hydrogen or covalent) to an edge. jViz.RNA then uses repulsion and attraction forces between all nucleotides to calculate the position and movement. This process continues until the forces reach an equilibrium for all nucleotides. Users can still move the individual nucleotides, and by doing so interact with the RNA structure. Mapping each nucleotide into a vertex creates a graph with a high number of vertexes and edges. As such, we denote this method as constructing a *detailed graph*.

A software similar to jViz.RNA, Forna, also uses a detailed graph representation in order to construct a dynamic RNA model. All nucleotides and chemical bonds are translated into vertexes and edges, and an automatic layout is constructed utilizing attraction and repulsion forces, as well as invisible "helper vertexes" which aid in the improvement of loop appearance. Like the previous three software tools, Forna allows for users to interact with the constructed dynamic model.

Of the four software tools mentioned, the approach employed by jViz.RNA and Forna relies mostly on the program to produce the layout, rather than require user intervention. This approach makes the production of RNA images less involved for the user, due to very little, if any, overlap even for large molecules. However, both Forna and jViz.RNA require more computation time for structure layout than PseudoViewer or VARNA. For very large structures of over 900nt, waiting for the automatic layout can be very inconvenient, and interaction with the structure becomes problematic as the structure’s movement becomes delayed. In addition, current jViz.RNA output contains some inconsistencies with regards to current accepted RNA visualization guidelines. The following section discusses these shortcomings in more detail.

### Motivation

**Fig. 1:**
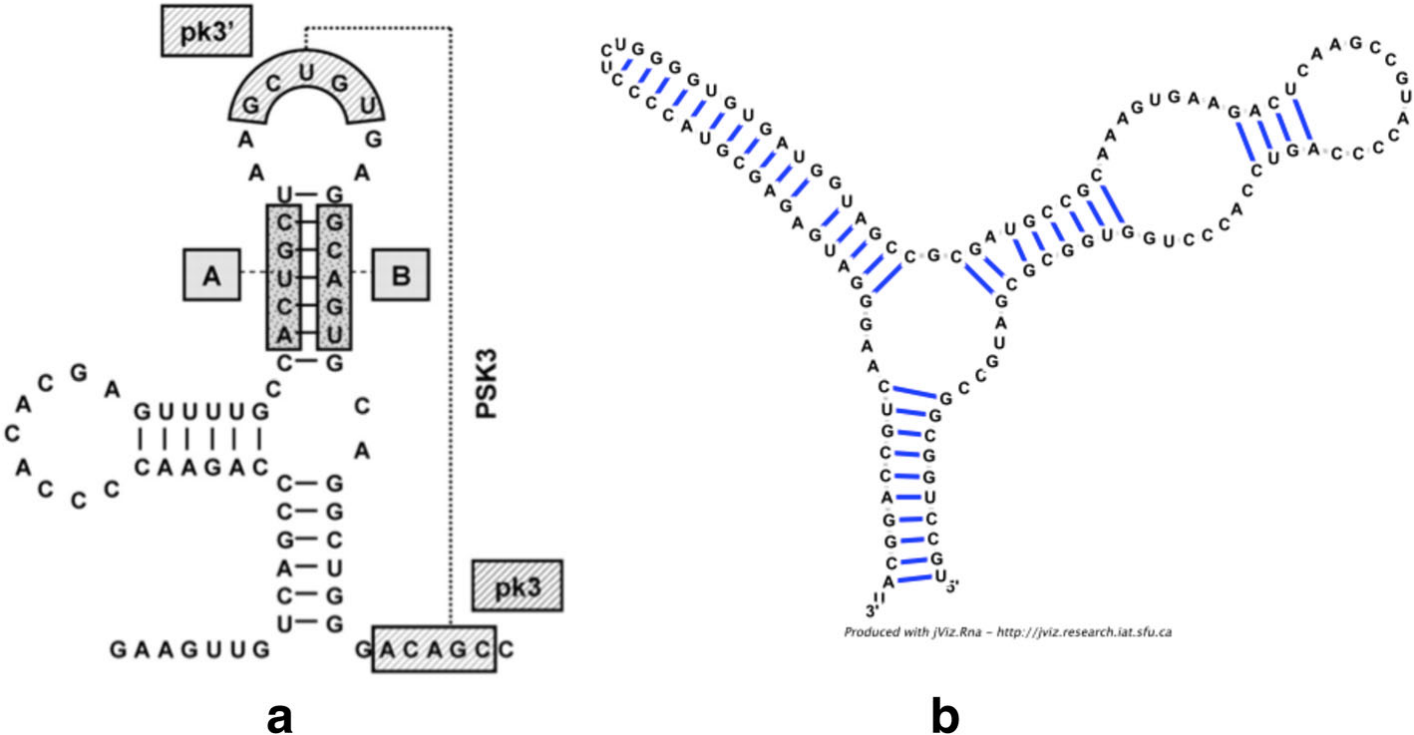
The visualization differences observed in jViz.RNA compared to an RNA image which highlights RNA visualization norms in literature. **a** The Yellow Fever Virus 3’ Untranslated Region (UTR). Image taken from [39] and used with permission, (**b**) An example produced by jViz.RNA of the *S. cervisiae* 5s ribosomal RNA (rRNA) (accession X67579)

While the current implementation for jViz.RNA produces dynamic RNA models that display secondary structure elements very well, the simple graph representation described presents some visual shortcomings. Most RNA images found in the literature follow several visualization norms, and two such norms that are very important are that stems are drawn such that the distance between base-pairs is consistent across the stem, and that loops are drawn as circular elements wherever they occur in the structure. Figure 1 demonstrates such limitations of jViz.RNA. While Fig. 1 a contains stems with consistent base-pair distances and loops which are clearly distinguishable by their circular nature, Fig. 1 b contains stems where the base pair distance varies across the stem, and some loops that are not immediately visible (such as the loop at the end of the left stem, and the bulge loop near the beginning of the right stem substructure).

The main motivation for the work presented in this manuscript was to address the visual shortcomings presented by the current algorithm employed in jViz.RNA, as well as improvements in the run-time performance. The objectives of this work were:

- To create an RNA representation which was more consistent with existing RNA visual conventions, such as round loops and equidistant base pairs.
- To design an automatic layout algorithm which works with the new representation, while introducing noticeable speed-ups to that automatic layout algorithm, and reduce overlap of structural elements.

Both objectives address important areas for improvement in jViz.RNA. While currently the resulting RNA molecule is laid out very clearly, personal feedback from RNA researchers, as well as visual comparison with other software, demonstrates that it ignores several visualization conventions such as the shape of loops and stems. This visualization pattern makes it more difficult to share informative images and diagrams about RNA, as the resulting visualization is not in the format most RNA scientists expect.

Another challenge the current setup faces is its time complexity. Calculating the attraction force for each nucleotide *n*_*i*_ is in *O*(1) since there are at most three nucleotides bonded to it, so the total calculation time for all the nucleotides’ attraction forces is in *O*(*N*) at each iteration (where *N* is the number of nucleotides in the RNA molecule). However, calculating the repulsion forces for each nucleotide is in *O*(*N*) since it must account for the repulsion of all other nucleotides, so the total calculation time for all the nucleotides’ repulsing forces is in *O*(*N* ^2^). This run-time can make the automatic layout algorithm perform slowly for large RNA structures such as 16S ribosomal RNA. As such, the work presented in this manuscript was set to implement the improvements while reducing the run-time required for the RNA structures to stabilize. In order to achieve this two-pronged effect, the method by which jViz.RNA builds the graph to represent the RNA structure had to be modified; the simple graphs built were replaced by a more compressed representation of the RNA molecule, named *compressed graphs*.

The work in this manuscript is divided into three main sections. First, we discuss how the RNA molecule can be mapped into a graph with a smaller number of vertexes, a compressed graph, similar to the one used by VARNA. Secondly, we profile the performance of the original version of jViz.RNA against the performance obtained by employing the reduced graph. Third and finally, a superior method for calculating movement is presented and profiled against the original movement calculation method.

## Methods

### Language, implementation, and system

The code presented in this manuscript was implemented using Java 6.0. The Swing library was used for the graphical component of the code. Measurements were all taken on a PC with an Intel Core i7-4790 3.60 GHz processor, running Ubuntu 14.04.5 LTS.

### Constructing and manipulating the new graph

**Fig. 2:**
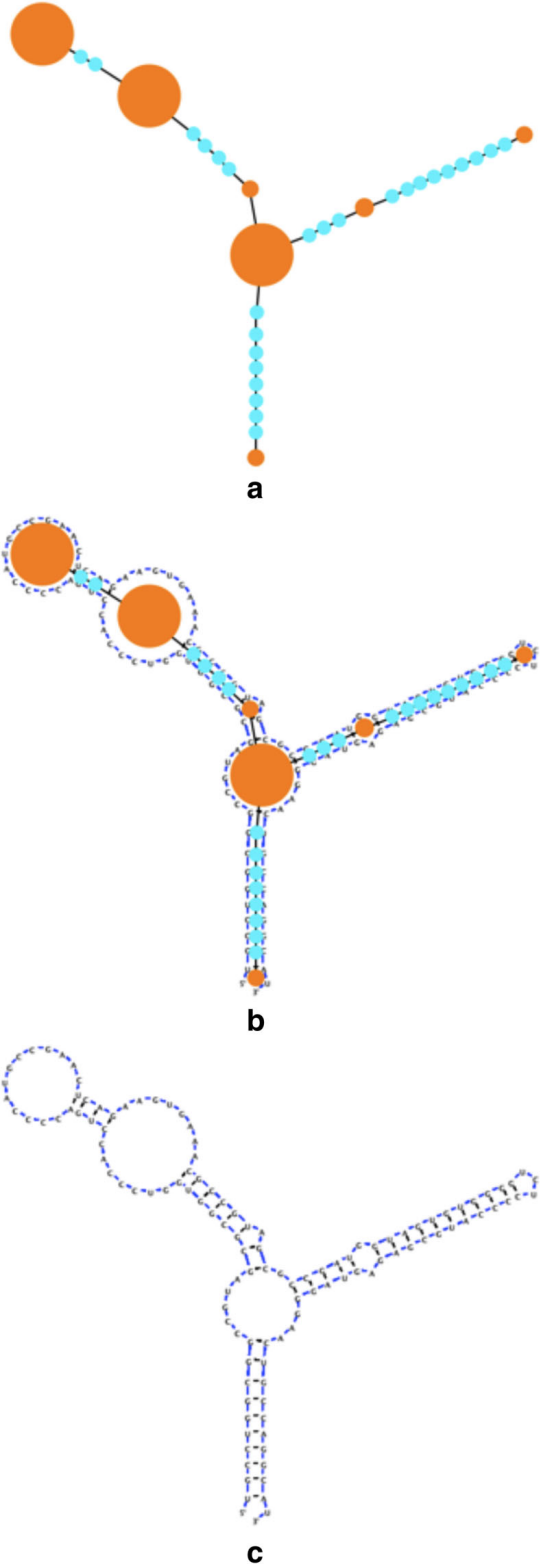
The compressed graph mapped to an RNA structure. **a** The main RNA elements are compressed into vertexes where each vertex represents an RNA loop element, or a stem base pair. **b** The nucleotides belonging to each RNA element are drawn on top of the underlying RNA compressed graph. **c** The resulting RNA representation contains less vertexes than there are nucleotides (in this case 120 nucleotides versus 34 vertexes), and a more familiar visual layout

One intuitive method of decreasing the run-time for the algorithm mentioned is to decrease the number of vertexes simulated. Since the run-time for the algorithm at every iteration is in order of *O*(*N* ^2^), decreasing the number of vertexes would theoretically have a tremendous effect on reducing the run-time. However, the mapping of the structure into vertexes and edges must be done in a way that still produces visually pleasing layouts and RNA diagrams. Inspired by VARNA, we have employed a representation which maps each RNA loop, as well as every stem base pair, to a vertex, and connects those via edges. (Fig. 2).

Furthermore, we have implemented the system in such a way that repulsion only occurs between loops, and not base pairs. Since the repulsion step is the main time consuming step of each iteration (having run time in order of *O*(*N* ^2^)), decreasing the number of participating vertexes in the repulsion interaction should greatly reduce the run time of the algorithm. Constructing the system in such a way that only loops experience repulsion ensures that loops will be pushed away from each other, thus not intersecting each other. In theory, this should aid the structure in adopting a final layout that has minimal or no intersection of any structural elements.

**Fig. 3:**
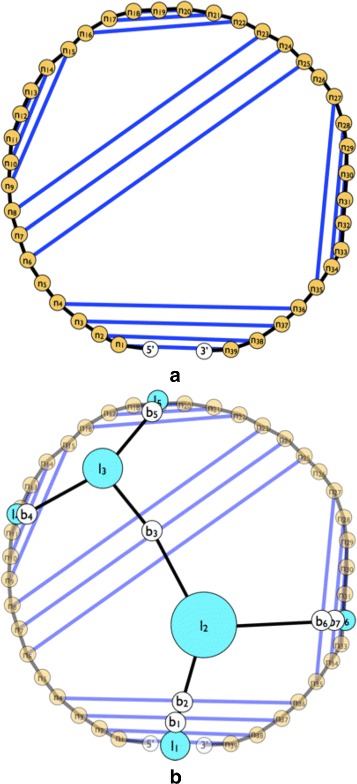
The initial vertex layout process demonstrated using a sample theoretical RNA molecule. *l*_1_, *l*_2_, *l*_3_, *l*_4_, *l*_5_, and *l*_6_ represent loops while *b*_1_, *b*_2_, *b*_3_, *b*_4_, *b*_5_, *b*_6_, and *b*_7_ represent base pairs. The loops and base pair vertexes are connected via *black* edges. **a** The RNA nucleotides are first laid out in a *circle*. **b** Each set of nucleotides has its average position calculated, and the vertex corresponding to that set is placed in that average position. Following this step, the iterative process of stabilization begins

At the initial step of the simulation, the RNA graphs are placed in a naive initial layout which is inspired from a circular representation of the RNA molecule (Fig. 3). Then, an iterative process begins in which the structure is slowly brought to a stable position by Newtonian inspired spring and repulsion forces.

### jViz.RNA and the Newtonian model

Originally jViz.RNA mapped the RNA structure into a detailed graph, *G*={*V,E*}. In the detailed graph representation, each nucleotide is a vertex *v*∈*V*, and each chemical bond corresponds to an edge *e*∈*E*. The entire structure is initially laid out in a circle, and an iterative process designed to move the structure into a stable layout begins. In this paper, we employ the ***compressed graph*** representation, where each structural element (loops and base pairs) is a vertex *v*∈*V*, and the edges are graph elements which connect the different structure elements (Fig. 2 b).

For the purposes of the following sections, the notation $\vec {P}$ will be used to denote the positions of the different vertexes which represent the RNA structures, and $\vec {P}^{i}_{n,k}$ will be used to denote the position of vertex *i* (since there are many vertexes), at time-step *n* (since the simulation operates in discrete time-steps), during the *k*-th Newton iteration (since a portion of the experiments in this paper use Newton’s method for converging on the position of vertex *i* at time-step *n*).

The most basic computation done at each iteration is the unit vector function, $\vec {U} (\vec {P}^{i}, \vec {P}^{j})$. This function calculates the unit vector pointing from point $\vec {P}^{j}$ (the position of vertex *j,v* ^*j*^) to point $\vec {P}^{i}$ (the position of vertex *i,v* ^*i*^) as follows:

1 $$ \vec{U} \left(\vec{P}^{i}, \vec{P}^{j}\right) = \frac{\vec{P}^{i}-\vec{P}^{j}}{\left|\vec{P}^{i}-\vec{P}^{j}\right|}  $$

In each iteration, each vertex moves based on two forces: repulsion and attraction. The repulsion forces each vertex *v* ^*i*^ experiences from vertex *v* ^*j*^ can be described as:

2 $$ \vec{R}\left(\vec{P}^{i}, \vec{P}^{j}\right) = \frac{G}{\left|\vec{P}^{i}-\vec{P}^{j}\right|^{2}} \times \vec{U} \left(\vec{P}^{i}, \vec{P}^{j}\right)  $$

where $\vec {U}(\vec {P}^{i}, \vec {P}^{j})$ is the unit vector function showing the direction from vertex *v* ^*j*^ to vertex *v* ^*i*^, and *G* is a coefficient to control the size of the force experienced. The attraction forces each vertex *v* ^*i*^ experiences from vertex *v* ^*j*^ can be described as:

3 $$ \vec{A}\left(\vec{P}^{i}, \vec{P}^{j}\right) = K \times \left[\vec{P}^{j}-\vec{P}^{i} + \left(r_{ideal}\times \vec{U} \left(\vec{P}^{i}, \vec{P}^{j}\right)\right)\right]   $$

where $\vec {U} (\vec {P}^{i}, \vec {P}^{j})$ is again the unit vector function, *K* is an attraction coefficient to control for the size of the force, and *r*_*ideal*_ is the ideal desired distance between the vertexes *v* ^*i*^ and *v* ^*j*^.

The iterative process stops when the forces for all vertexes have reached equilibrium, or when for all vertexes {*v* ^*i*^|1≤*i*≤*N*} the following holds:

4 $$ \forall v^{i}, \left| \sum\limits_{v^{j} \in L,j\ne i} \vec{R}\left(\vec{P}^{i}, \vec{P}^{j}\right) + \sum\limits_{v^{j} \in C^{i}} \vec{A}\left(\vec{P}^{i}, \vec{P}^{j}\right)\right| \le \epsilon  $$

Where *C* ^*i*^ is the set of all vertexes connected to vertex *v* ^*i*^ (in other words, *v* ^*j*^∈*C* ^*i*^ iff there is an edge between *v* ^*j*^ and *v* ^*i*^), and *L* is the set of all loops (that is, *v* ^*j*^∈*L* iff *v* ^*j*^ is a vertex representing a loop).

That is to say, the iterative process stops when the sum of the forces acting on the vertexes is smaller than *ε*. Setting *ε*=0 will force the simulation to continue to calculate until the forces are perfectly at odds, but setting a small value for *ε* allows the layout algorithm to stop sooner when achieving a stable structure. *ε* as such, controls the degree of stability required before simulation of the structure’s movement stops. In this work, we chose to explore two methods of implementing the physics based RNA model: The Forward Euler method, and the Backward Euler method. The two methods make it possible to evaluate the movement of the vertexes. However, the latter is more numerically stable than the former, and allows for greater time steps and faster visualizations, as well as a more stable user interaction experience.

### The forward Euler method

The Euler method is a first-order integration method which belongs to a larger class called the Runge-Kutta methods (most famous being the fourth-order method [31]). The simplest version is called the Forward Euler Method [32]. This method of calculating each time step can be expressed in the following manner:

5 $$ \begin{aligned} t_{n+1} & = t_{n} + \Delta t\\ \vec{P}^{i}_{n+1} & = \vec{P}^{i}_{n} + \Delta tf(t_{n}, \vec{P}^{i}_{n}) \end{aligned}  $$

which means the time *t*_*n*_ is advanced by the time-step *Δ* *t* and then the position of the vertex *v* ^*i*^ is updated based on the size of the time-step, and the current behaviour of the particle, *f* (which is usually a function which depends on the particle’s current state and/or the time).

When applied to the movement of the RNA vertexes, the Forward Euler method can be written as:

6 $$ \begin{array}{lcl} t_{n+1} & = & t_{n} + \Delta t\\ f(t_{n}, \vec{P}^{i}_{n}) & = & \sum_{v^{j} \in L,j\ne i} \vec{R}\left(\vec{P}^{i}_{n}, \vec{P}^{j}_{n}\right) + \sum\limits_{v^{j} \in C^{i}} \vec{A}\left(\vec{P}^{i}_{n}, \vec{P}^{j}_{n}\right)\\ \vec{P}^{i}_{n+1} & = & \vec{P}^{i}_{n} + \Delta tf\left(t_{n}, \vec{P}^{i}_{n}\right) \end{array}   $$

where in this case, the behaviour of the particle with regard to its position is the sum of repulsion ($\vec {R}$) forces (over all loops *v* ^*j*^∈*L*, where *L* is the set of all loops) and attraction ($\vec {A}$) forces acting on the particle.

Since base pair vertexes do no participate in the repulsion step, the expression for a base pair vertex’s Forward Euler implementation will be:

7 $$ \begin{array}{lcl} t_{n+1} & = & t_{n} + \Delta t\\ f\left(t_{n}, \vec{P}^{i}_{n}\right) & = & \sum_{v^{j} \in C^{i}} \vec{A}\left(\vec{P}^{i}_{n}, \vec{P}^{j}_{n}\right)\\ \vec{P}^{i}_{n+1} & = & \vec{P}^{i}_{n} + \Delta tf\left(t_{n}, \vec{P}^{i}_{n}\right) \end{array}   $$

However, since the implementation of this expression is trivially similar to the expression in (6), the remainder of this text will focus on that expression, with the implications for the expression in (7) being omitted for brevity.

The main drawback presented by the Forward Euler method is its numerical instability. Simply put, when the time-step *Δ* *t* is too long, or the coefficients which control the simulation become too large, the simulation does not stabilize into an equilibrium. In fact, it can become increasingly unstable. The solution to this drawback lies in the implementation of the Backward Euler method, which takes this instability into account.

### The backward Euler method

Much like the Forward Euler method is described as explicit, there is an implicit Euler method; the Backward Euler method. Generally, it is defined as:

8 $$ \begin{array}{lcl} t_{n+1} & = & t_{n} + \Delta t\\ \vec{P}^{i}_{n+1} & = & \vec{P}^{i}_{n} + \Delta tf\left(t_{n}, \vec{P}^{i}_{n+1}\right) \end{array}   $$

where $f(t_{n}, \vec {P}^{i}_{n+1})$ is again a function that describes the movement of the object. Notice it is very similar to the explicit method, but the term $\vec {P}^{i}_{n+1}$ appears on both sides of the equation. As a result, finding $\vec {P}^{i}_{n+1}$ is no longer a simple issue of updating the timestep, but it is that of solving for it algebraically.

In the case of the current simulation, the Backward Euler method would yield the following expression:

9 $$ \begin{array}{lcl} t_{n+1} & = & t_{n} + \Delta t\\ f\left(t_{n}, \vec{P}^{i}_{n+1}\right) & = & \sum_{v^{j} \in L,j\ne i} \vec{R}\left(\vec{P}^{i}_{n+1}, \vec{P}^{j}_{n}\right) \! +\!\!\! \sum\limits_{v^{j} \in C^{i}} \vec{A}\left(\vec{P}^{i}_{n+1}, \vec{P}^{j}_{n}\right)\\ \vec{P}^{i}_{n+1} & = & \vec{P}^{i}_{n} + \Delta tf\left(t_{n}, \vec{P}^{i}_{n+1}\right) \end{array}   $$

which becomes a fairly difficult equation to solve for $\vec {P}^{i}_{n+1}$ directly. Instead, an approximation is used to solve for $\vec {P}^{i}_{n+1}$.

### Applying Newton’s method to solve the Backward Euler expression

The expression in (9) can be rearranged to produce the following equation:

10 $$ {\begin{aligned} \vec{P}^{i}_{n+1} = \vec{P}^{i}_{n} + \Delta t\left[\sum\limits_{v^{j} \in L,j\ne i} \vec{R}\left(\vec{P}^{i}_{n+1}, \vec{P}^{j}_{n}\right) + \sum\limits_{v^{j} \in C^{i}} \vec{A}\left(\vec{P}^{i}_{n+1}, \vec{P}^{j}_{n}\right)\right] \end{aligned}}  $$

which can be rewritten as:

11 $$ {\begin{aligned} \vec{F}\left(\vec{P}^{i}_{n+1}\right)&= \vec{0} = - \vec{P}^{i}_{n+1} + \vec{P}^{i}_{n}\\ &\quad+ \Delta t\left[\sum\limits_{v^{j} \in L,j\ne i} \vec{R}\left(\vec{P}^{i}_{n+1}, \vec{P}^{j}_{n}\right) + \sum\limits_{v^{j} \in C^{i}} \vec{A}\left(\vec{P}^{i}_{n+1}, \vec{P}^{j}_{n}\right)\right] \end{aligned}}  $$

meaning the solution for $\vec {P}^{i}_{n+1}$ is the root of the function $\vec {F}(\vec {P}^{i}_{n+1})$. While it may be difficult to solve for the root directly, Newton’s method offers an approach for approximating the root of the vector function $\vec {F}\left (\vec {P}^{i}_{n+1}\right)$ [33].

#### Defining the vector function’s components

As outlined in [33], it is necessary to define each of the components in $\vec {F}$ individually so that their derivatives can then be found with respect to each of the variables. In the case of the RNA simulation, the function $\vec {F}$ contains only two components; *f*_*x*_ and *f*_*y*_ which are each defined as:

12 $$  \begin{array}{lcl} f_{x}\left(\vec{P}^{i}_{n+1}\right) & = & - x^{i}_{n+1} + x^{i}_{n} + \Delta t\left[\sum_{v^{j} \in L,j\ne i} R_{x}\left(\vec{P}^{i}_{n+1}, \vec{P}^{j}_{n}\right) \right.\\ &&\left.+ \sum_{v^{j} \in C^{i}} A_{x}\left(\vec{P}^{i}_{n+1}, \vec{P}^{j}_{n}\right)\right]\\ f_{y}\left(\vec{P}^{i}_{n+1}\right) & = & - y^{i}_{n+1} + y^{i}_{n} + \Delta t\left[\sum_{v^{j} \in L,j\ne i} R_{y}\left(\vec{P}^{i}_{n+1}, \vec{P}^{j}_{n}\right)\right.\\ &&\left.+ \sum_{v^{j} \in C^{i}} A_{y}\left(\vec{P}^{i}_{n+1}, \vec{P}^{j}_{n}\right)\right] \end{array}   $$

This definition requires both $\vec {R}$ and $\vec {A}$ (as well as $\vec {U}$) to be defined in terms of their *x* and *y* components as:

13 $$ {\begin{aligned} \begin{array}{lcl} R_{x}\left(\vec{P}^{i}_{n+1}, \vec{P}^{j}_{n}\right) & = & \frac{G}{\left(x^{i}_{n+1} - x^{j}_{n}\right)^{2}+\left(y^{i}_{n+1} - y^{j}_{n}\right)^{2}} \times U_{x} \left(\vec{P}^{i}_{n+1}, \vec{P}^{j}_{n}\right)\\ R_{y}\left(\vec{P}^{i}_{n+1}, \vec{P}^{j}_{n}\right) & = & \frac{G}{\left(x^{i}_{n+1} - x^{j}_{n}\right)^{2}+\left(y^{i}_{n+1} - y^{j}_{n}\right)^{2}} \times U_{y} \left(\vec{P}^{i}_{n+1}, \vec{P}^{j}_{n}\right)\\ A_{x}\left(\vec{P}^{i}_{n+1}, \vec{P}^{j}_{n}\right) & = & K \times \left[x^{j}_{n}-x^{i}_{n+1} + \left(r_{ideal}\times U_{x} \left(\vec{P}^{i}_{n+1}, \vec{P}^{j}_{n}\right)\right.\right]\\ A_{y}\left(\vec{P}^{i}_{n+1}, \vec{P}^{j}_{n}\right) & = & K \times \left[y^{j}_{n}-y^{i}_{n+1} + \left(r_{ideal}\times U_{y} \left(\vec{P}^{i}_{n+1}, \vec{P}^{j}_{n}\right)\right.\right]\\ U_{x} \left(\vec{P}^{i}_{n+1}, \vec{P}^{j}_{n}\right) & = & \frac{x^{i}_{n+1}-x^{j}_{n}}{\sqrt{\left.x^{i}_{n+1} - x^{j}_{n}\right)^{2}+\left(y^{i}_{n+1} - y^{j}_{n}\right)^{2}}}\\ U_{y} \left(\vec{P}^{i}_{n+1}, \vec{P}^{j}_{n}\right) & = & \frac{y^{i}_{n+1}-y^{j}_{n}}{\sqrt{\left.x^{i}_{n+1} - x^{j}_{n}\right)^{2}+\left(y^{i}_{n+1} - y^{j}_{n}\right)^{2}}} \end{array} \end{aligned}}   $$

#### Finding the components’ derivatives

In order to apply Newton’s method to the RNA model, the Jacobian matrix *D* of the vector function $\vec {F}$ needs to be defined. In order to do so, expressions for all partial derivatives of the components in Eqs. (12) - (13) need to be defined, where each component has two partial derivatives; with respect to $x^{i}_{n+1}$ and with respect to $y^{i}_{n+1}$. The derivation of each component’s partial derivatives is quite long and is not the main focus of this article. Therefore, for brevity purposes, the individual derivatives are outlined in the set of Eqs. (14)-(17):

14 $$ {\begin{aligned} \begin{array}{lcl} \frac{\delta f_{x}}{\delta x^{i}_{n+1}}\left(\vec{P}^{i}_{n+1}\right) & = & - 1+ \Delta t\left[\sum_{v^{j} \in L,j\ne i} \frac{\delta R_{x}}{\delta x^{i}_{n+1}}\left(\vec{P}^{i}_{n+1}, \vec{P}^{j}_{n}\right) \right.\\ &&\left.+ \sum_{v^{j} \in C^{i}} \frac{\delta A_{x}}{\delta x^{i}_{n+1}}\left(\vec{P}^{i}_{n+1}, \vec{P}^{j}_{n}\right)\right]\\ \frac{\delta f_{x}}{\delta y^{i}_{n+1}}\left(\vec{P}^{i}_{n+1}\right) & = & \Delta t\left[\sum_{v^{j} \in L,j\ne i} \frac{\delta R_{x}}{\delta y^{i}_{n+1}}\left(\vec{P}^{i}_{n+1}, \vec{P}^{j}_{n}\right)\right.\\ &&\left.+ \sum_{v^{j} \in C^{i}} \frac{\delta A_{x}}{\delta y^{i}_{n+1}}\left(\vec{P}^{i}_{n+1}, \vec{P}^{j}_{n}\right)\right]\\ \frac{\delta f_{y}}{\delta x^{i}_{n+1}}\left(\vec{P}^{i}_{n+1}\right) & = & \Delta t\left[\sum_{v^{j} \in L,j\ne i} \frac{\delta R_{y}}{\delta x^{i}_{n+1}}\left(\vec{P}^{i}_{n+1}, \vec{P}^{j}_{n}\right)\right.\\ &&\left.+ \sum_{v^{j} \in C^{i}} \frac{\delta A_{y}}{\delta x^{i}_{n+1}}\left(\vec{P}^{i}_{n+1}, \vec{P}^{j}_{n}\right)\right]\\ \frac{\delta f_{y}}{\delta y^{i}_{n+1}}\left(\vec{P}^{i}_{n+1}\right) & = & -1 + \Delta t\left[\sum_{v^{j} \in L,j\ne i} \frac{\delta R_{y}}{\delta y^{i}_{n+1}}\left(\vec{P}^{i}_{n+1}, \vec{P}^{j}_{n}\right)\right.\\ &&\left.+ \sum_{v^{j} \in C^{i}} \frac{\delta A_{y}}{\delta y^{i}_{n+1}}\left(\vec{P}^{i}_{n+1}, \vec{P}^{j}_{n}\right)\right] \end{array} \end{aligned}}   $$

15 $$ {\begin{aligned} \begin{array}{rcl} r &= & \left(x^{i}_{n+1} - x^{j}_{n}\right)^{2}+\left(y^{i}_{n+1} - y^{j}_{n}\right)^{2}\\ \frac{\delta R_{x}}{\delta x^{i}_{n+1}}\left(\vec{P}^{i}_{n+1}, \vec{P}^{j}_{n}\right) & = & \left\{\left[\frac{\delta U_{x}}{\delta x^{i}_{n+1}}\left(\vec{P}^{i}_{n+1}, \vec{P}^{j}_{n}\right) \times \frac{G}{r}\right]\right.\\ &&\left.- \left[\frac{2G\left(x^{i}_{n+1} - x^{j}_{n}\right)}{r^{2}} \times U_{x} \left(\vec{P}^{i}_{n+1}, \vec{P}^{j}_{n}\right)\right]\right\} \\ \frac{\delta R_{x}}{\delta y^{i}_{n+1}}\left(\vec{P}^{i}_{n+1}, \vec{P}^{j}_{n}\right) & = & \left\{\left[\frac{\delta U_{x}}{\delta y^{i}_{n+1}}\left(\vec{P}^{i}_{n+1}, \vec{P}^{j}_{n}\right) \times \frac{G}{r}\right]\right.\\ &&\left.- \left[\frac{2G\left(y^{i}_{n+1} - y^{j}_{n}\right)}{r^{2}} \times U_{x} \left(\vec{P}^{i}_{n+1}, \vec{P}^{j}_{n}\right)\right]\right\}\\ \frac{\delta R_{y}}{\delta x^{i}_{n+1}}\left(\vec{P}^{i}_{n+1}, \vec{P}^{j}_{n}\right) & = & \left\{\left[\frac{\delta U_{y}}{\delta x^{i}_{n+1}}\left(\vec{P}^{i}_{n+1}, \vec{P}^{j}_{n}\right) \times \frac{G}{r}\right]\right.\\ &&\left.- \left[\frac{2G\left(x^{i}_{n+1} - x^{j}_{n}\right)}{r^{2}} \times U_{y} \left(\vec{P}^{i}_{n+1}, \vec{P}^{j}_{n}\right)\right]\right\}\\ \frac{\delta R_{y}}{\delta y^{i}_{n+1}}\left(\vec{P}^{i}_{n+1}, \vec{P}^{j}_{n}\right) & = & \left\{\left[\frac{\delta U_{y}}{\delta y^{i}_{n+1}}\left(\vec{P}^{i}_{n+1}, \vec{P}^{j}_{n}\right) \times \frac{G}{r}\right]\right.\\ &&\left.- \left[\frac{2G\left(y^{i}_{n+1} - y^{j}_{n}\right)}{r^{2}} \times U_{y} \left(\vec{P}^{i}_{n+1}, \vec{P}^{j}_{n}\right)\right]\right\} \end{array} \end{aligned}}   $$

16 $$ {\begin{aligned} \begin{array}{lcl} \frac{\delta A_{x}}{\delta x^{i}_{n+1}}\left(\vec{P}^{i}_{n+1}, \vec{P}^{j}_{n}\right) & = & K \times \left[-1 + \left(r_{ideal}\times \frac{\delta U_{x}}{\delta x^{i}_{n+1}} \left(\vec{P}^{i}_{n+1}, \vec{P}^{j}_{n}\right)\right)\right]\\ \frac{\delta A_{x}}{\delta y^{i}_{n+1}}\left(\vec{P}^{i}_{n+1}, \vec{P}^{j}_{n}\right) & = & K \times \left(r_{ideal}\times \frac{\delta U_{x}}{\delta y^{i}_{n+1}} \left(\vec{P}^{i}_{n+1}, \vec{P}^{j}_{n}\right)\right)\\ \frac{\delta A_{y}}{\delta x^{i}_{n+1}}\left(\vec{P}^{i}_{n+1}, \vec{P}^{j}_{n}\right) & = & K \times \left(r_{ideal}\times \frac{\delta U_{y}}{\delta x^{i}_{n+1}} \left(\vec{P}^{i}_{n+1}, \vec{P}^{j}_{n}\right)\right)\\ \frac{\delta A_{y}}{\delta y^{i}_{n+1}}\left(\vec{P}^{i}_{n+1}, \vec{P}^{j}_{n}\right) & = & K \times \left[-1 + \left(r_{ideal}\times \frac{\delta U_{y}}{\delta y^{i}_{n+1}} \left(\vec{P}^{i}_{n+1}, \vec{P}^{j}_{n}\right)\right)\right]\\ \end{array} \end{aligned}}   $$

17 $$ \begin{array}{rcl} r &= & \sqrt{\left(x^{i}_{n+1} - x^{j}_{n}\right)^{2}+\left(y^{i}_{n+1} - y^{j}_{n}\right)^{2}}\\ \frac{\delta U_{x}}{\delta x^{i}_{n+1}} \left(\vec{P}^{i}_{n+1}, \vec{P}^{j}_{n}\right) & = & \frac{\left(y^{i}_{n+1}-y^{j}_{n}\right)^{2}}{r^{3}}\\ \frac{\delta U_{x}}{\delta y^{i}_{n+1}} \left(\vec{P}^{i}_{n+1}, \vec{P}^{j}_{n}\right) & = & \frac{-\left(y^{i}_{n+1}-y^{j}_{n}\right)\left(x^{i}_{n+1}-x^{j}_{n}\right)}{r^{3}}\\ \frac{\delta U_{y}}{\delta x^{i}_{n+1}} \left(\vec{P}^{i}_{n+1}, \vec{P}^{j}_{n}\right) & = & \frac{-\left(y^{i}_{n+1}-y^{j}_{n}\right)\left(x^{i}_{n+1}-x^{j}_{n}\right)}{r^{3}}\\ \frac{\delta U_{y}}{\delta y^{i}_{n+1}} \left(\vec{P}^{i}_{n+1}, \vec{P}^{j}_{n}\right) & = & \frac{\left(x^{i}_{n+1}-x^{j}_{n}\right)^{2}}{r^{3}} \end{array}   $$

and the matrix D is defined as:

$$D\left(\vec{P}^{i}_{n+1}\right)= \left[\begin{array}{cc} \frac{\delta f_{x}}{\delta x^{i}_{n+1}}\left(\vec{P}^{i}_{n+1}\right) & \frac{\delta f_{x}}{\delta y^{i}_{n+1}}\left(\vec{P}^{i}_{n+1}\right)\\ \frac{\delta f_{y}}{\delta x^{i}_{n+1}}\left(\vec{P}^{i}_{n+1}\right) & \frac{\delta f_{y}}{\delta y^{i}_{n+1}}\left(\vec{P}^{i}_{n+1}\right) \end{array}\right]. $$

#### Constructing the Newton step

Given the function $\vec {F}$ and the matrix D, progressively better estimates for the value of $\vec {P}^{i}_{n+1}$ can be found by applying the following Newton step:

18 $$ \vec{P}^{i}_{n+1,k+1} = \vec{P}^{i}_{n+1,k} - \vec{F}\left(\vec{P}^{i}_{n+1,k}\right)\times D^{-1}\left(\vec{P}^{i}_{n+1,k}\right)  $$

where $D^{-1}\left (\vec {P}^{i}_{n+1,k}\right)$ is the inverse matrix of $D\left (\vec {P}^{i}_{n+1,k}\right)$. That is, at every Newton step *k*+1, the value of both the function $\vec {F}$ and its components’ derivatives, encapsulated in the matrix *D* ^−1^, are evaluated at the point $\vec {P}^{i}_{n+1,k}$, that is, the point $\vec {P}^{i}_{n+1}$ from the previous Newton step. The initial estimate, $\vec {P}^{i}_{n+1,0}$ can be obtained by applying the Forward Euler. As more Newton steps are repeated, a better and better estimate for $\vec {P}^{i}_{n+1}$ emerges. However, each Newton step increases the run-time of each iteration of the algorithm. In general, each additional Newton step increases the run time of the physics based simulation by *O*(*L* ^2^) where *L* is the number of loops in the simulation.

### Experimental parameters and test-bed structures

For the purposes of these experiments, 17 RNA molecules were chosen from the RNA STRAND v2.0 database [34], and were run under two different configurations. The configurations and their parameters can be found in Table 1, while the structure details can be found in Table 2 ^1^. The structure lengths are given in “nt,” which stands for “nucleotides.”

**Table 1:** The parameters for the two experimental configurations

Configuration #	1	2
Movement update	Forward Euler	Backward Euler
K	10.0	10.0
G	0.01	0.01
Time-step (*Δ* *t*)	0.01	3.0
Minimal stablization movement (*ε*)	0.0001	0.3
Newton iterations	N/A	5

**Table 2:** The RNA structures chosen for comparison between the forward and backward Euler methods

#	RNA	Original	Length	Reference
	STRAND ID	ID	(nt)	
1	NDB_00051	PDB: 1VTQ	75	[40]
2	PDB_01255	PDB: 2R8S	159	[41]
3	PDB_01076	PDB: 2GO5	217	[42]
4	PDB_00985	PDB: 2CZJ	248	[43]
5	PDB_00528	PDB: 1KOG	304	[44]
6	PDB_00398	PDB:1FCW	380	[45]
7	PDB_01144	PDB: 2J37	408	[46]
8	SRP_00288	SRPDB: Sacc.cere._M28116	522	[47]
9	RFA_00829	Rfam: RF00551	551	[48]
10	CRW_00736	CRW: a.I2.c.N.tabacum.B.ND2	696	[35]
11	CRW_00731	CRW: a.I2.c.N.tabacum.A.trnI.i1	772	[35]
12	CRW_00757	CRW: a.I2.m.Z.mays.A.OX2.i1	912	[35]
13	CRW_00533	CRW: d.233.m.C.elegans	953	[35]
14	CRW_00540	CRW: d.233.m.L.terrestris	1279	[35]
15	CRW_00539	CRW: d.233.m.L.bleekeri	1333	[35]
16	CRW_00742	CRW: a.I2.m.A.aegerita.B.LSU.2059	1857	[35]
17	CRW_00534	CRW: d.233.m.C.eugametos	1915	[35]

Different time-steps were chosen for the different configurations (Table 1). Configuration 1 was assigned the highest time-steps it can support without losing stability. Configuration 2 can handle larger time steps, but the choice of time-step influences the choice for the number of Newton iterations (such that larger time steps required more Newton iterations to reach convergence). Therefore, a value of 3.0 was chosen to support satisfactory convergence within 5 Newton iterations.

Each structure was run 20 times and the CPU time of the run was measured until the structure stabilized (that is, until the large movement of any of its components was less than *ε*). The average run-time was calculated and plotted. If a structure’s stabilization process took more than 30 mins (1800 s) it was terminated and its stabilization time was taken as 1800 s.

### Improving the attraction force calculations

**Fig. 4:**
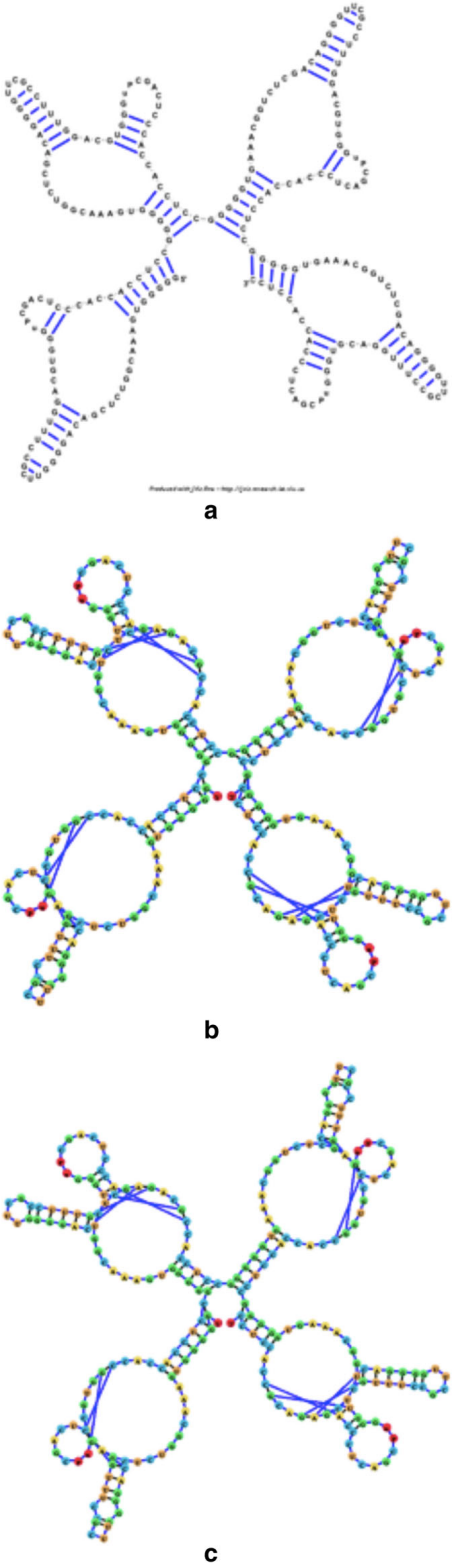
The visualization result obtained for the 248 nt RNA (RNA STRAND ID PDB_00985). **a** The visualization obtained with jViz’s detailed graph representation (employing the Forward Euler method). **b** The visualization obtained with jViz’s compressed graph representation and the Forward Euler method. **c** The visualization obtained with jViz’s compressed graph representation and the Backward Euler method

The system of forces described in the previous section allowed the RNA structure simulation to stabilize and present the RNA structural elements much better than the former jViz.RNA implementation (Fig. 4 a). However, the resulting stable layouts were not satisfactory due to the overlap artefacts created (Fig. 4 b–c). Stems would often overlap loops and would not stabilize into their correct position based on their connectivity to the loops. While a user could, in theory, address such a problem manually, we felt there is room for further improvements. In order to correct the overlap artefacts, a slight modification to the attraction force calculation was implemented.

**Fig. 5:**
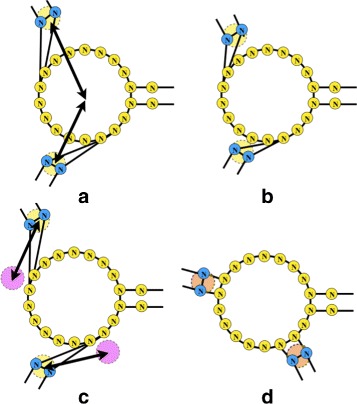
Implementing the ideal position attraction forces causes the stems to align with their ideal layout. **a** Originally, attraction forces were acting between base pairs, and the loops, attracting the centres of the vertexes directly. **b** The resulting layout contained artefacts of distorted stems, since base-pairs were unaware of their positions relative to loops. **c** The idealized attraction forces employ the ideal positions (*purple circles*) of the stems to attract the base-pair vertexes. **d** The resulting layout when employing the ideal positions is aware of the position stems should take relative to their parent loops

Originally, the attraction forces would apply attraction between the centres of two vertexes (Fig. 5 a–b). However, with a slight modification, each vertex can store the ideal positions for each stem protruding from it (Fig. 5 c). Using these ideal positions in the equation for $\vec {A}(P^{i}, P^{j})$ to move each vertex to its ideal position and orientation (Fig. 5 d). The resulting layouts prove to be much more visually appealing and containing much less overlap, especially for smaller RNA structures (Figs. 8, 9, 10, 11, 12 and 13).

## Results

### Comparison of jViz.RNA’s performance employing the forward and backward Euler methods

**Fig. 6:**
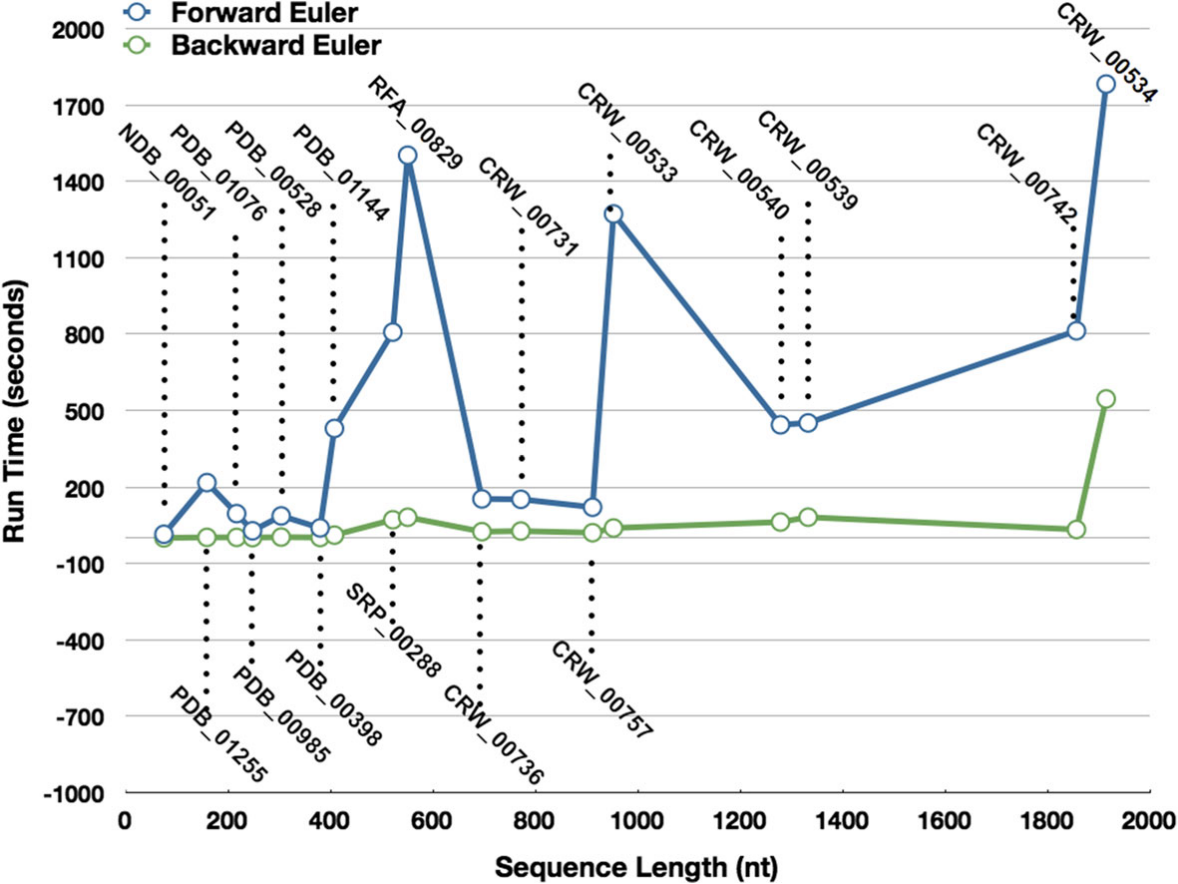
The Run-times (expressed in seconds) of jViz.RNA’s compressed graph representations employing both the Forward and Backward Euler methods

**Fig. 7:**
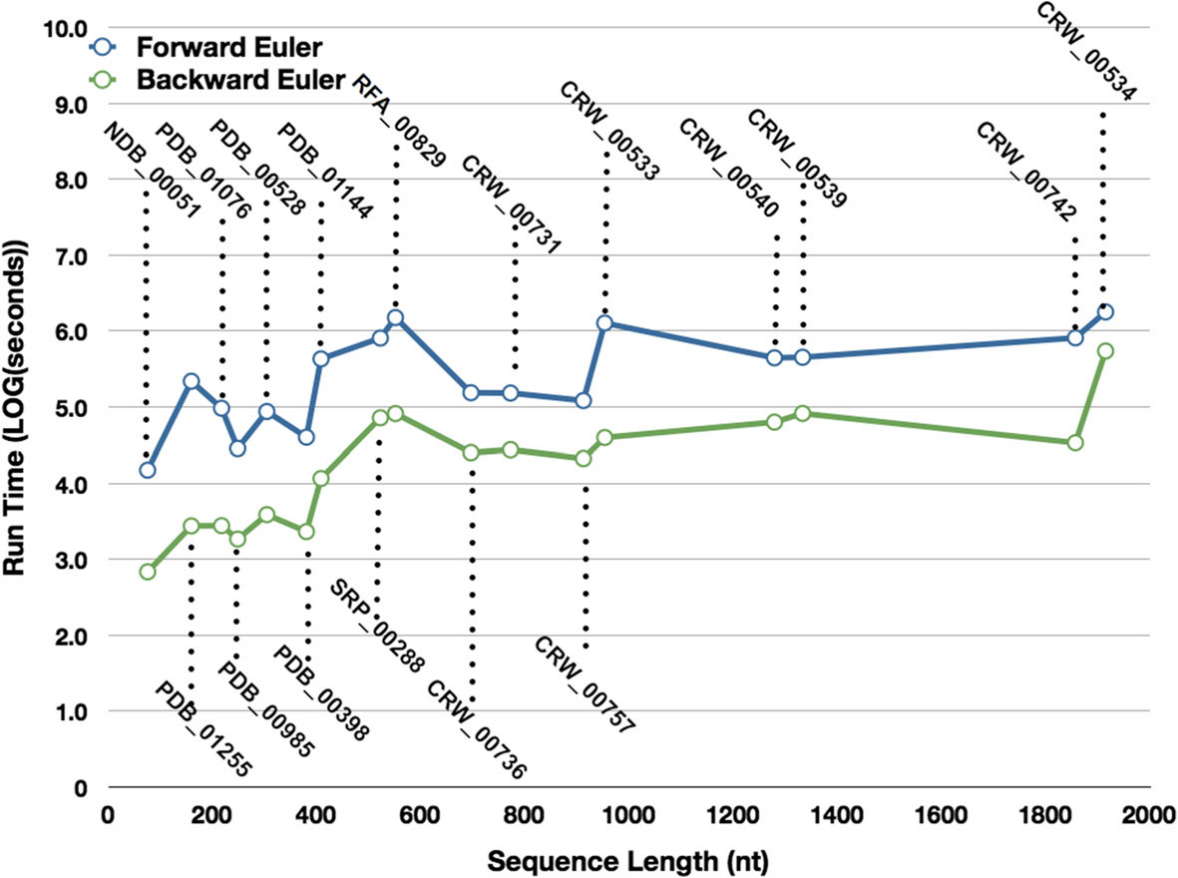
The Run-times (expressed in *log*_10_(seconds)) of jViz.RNA’s compressed graph representations employing both the Forward and Backward Euler methods

Figure 6 shows the run times of jViz.RNA when employing the Forward and Backward Euler method. As expected, since the Backward Euler method takes a much larger time step, the structures subject to the Backward Euler simulation converge to a stable layout much more rapidly than when subject to the Forward Euler. In fact, to truly appreciate the difference, the *log*_10_ of the run times was taken and plotted in Fig. 7. As can be seen, the run times of the Forward Euler method are often ≈100 times longer than the Backward Euler run times. Considering the fact that no structure was allowed longer than 1800 seconds to stabilize, it is fair to assume that under the current parameters of *K* and *G*, the difference in run time could have been even greater for some structures.

One would expect that the run-times would increase in a quadratic order to the number of nucleotides. However, while there is a general increase in run time with structure size, some small structures take longer than larger ones to stabilize. This observation points to the fact that the connectivity of the structure plays a very important role in its stabilization time. Overall, a structure X composed of 3 times as many nucleotides as structure Y would take longer to stabilize, but it may not be straightforward to deduce exactly how much longer. Even the number of vertexes in a given structure does not provide a good heuristic to calculating the difference in stabilization time for both the Forward and Backward Euler.

Despite the relative uncertainty in the relationship between a given structure’s run time and its size, there is a great deal of certainty that the Backward Euler proved superior when compared to the Forward Euler. First, it can produce stable layouts employing a time step 300 times larger than the Forward Euler method without losing stability. Second, it exhibits much faster run-time performance. As demonstrated in this work, some large structures may pose a challenge to a system which takes smaller time step since the topology of the structure itself dictates how long it will take to stabilize

### Visual comparison of the different algorithms

**Fig. 8:**
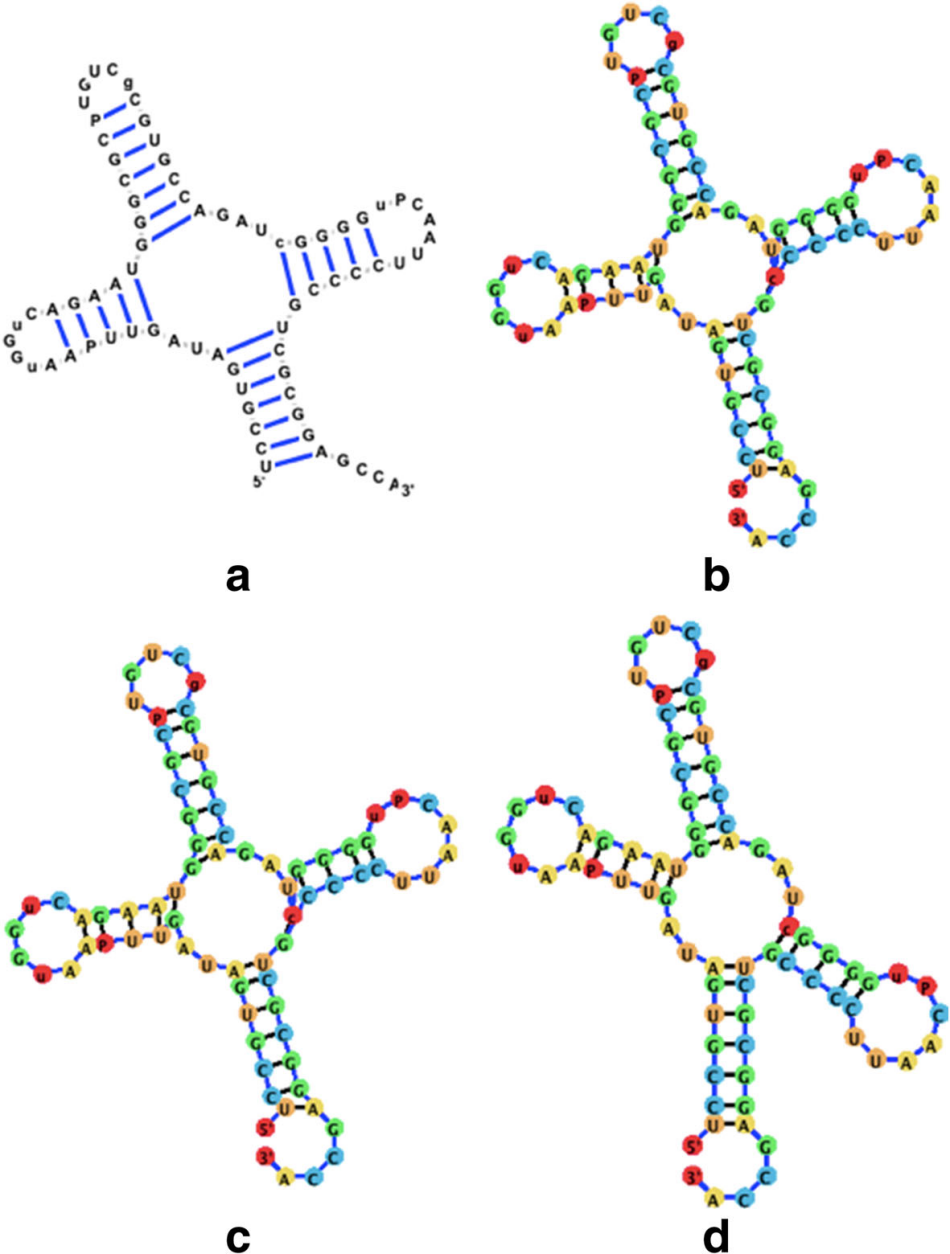
The visualization result obtained for the 75 nt RNA (RNA STRAND ID NDB_00051) utilizing: **a** The detailed graph representation (employing the Forward Euler method). **b** The compressed graph representation and the Forward Euler method. **c** The compressed graph representation and the Backward Euler method. **d** The compressed graph representation and the Backward Euler method while employing ideal positions attraction forces

**Fig. 9:**
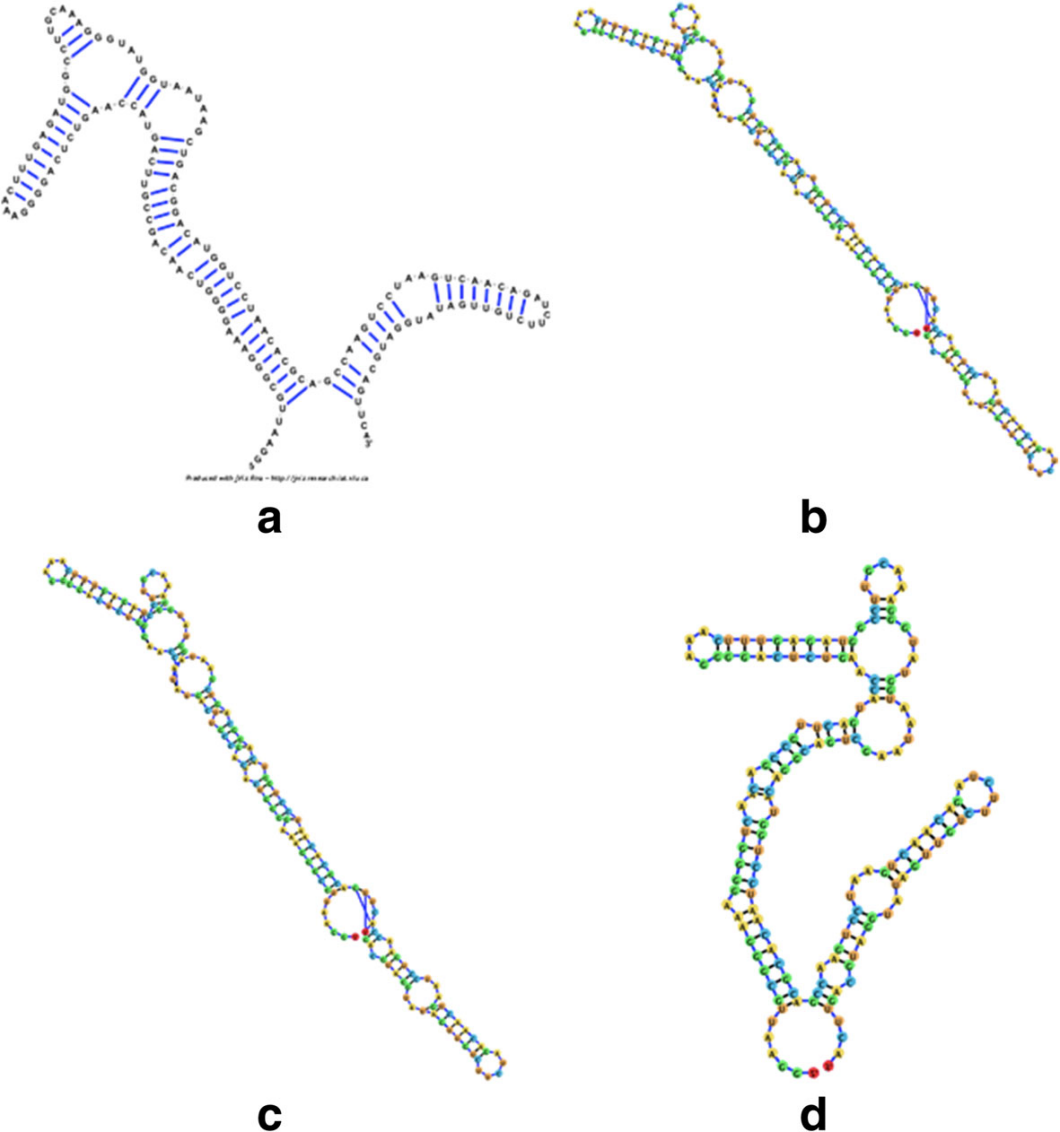
The visualization result obtained for the 159 nt RNA (RNA STRAND ID PDB_01255) utilizing: **a** The detailed graph representation (employing the Forward Euler method). **b** The compressed graph representation and the Forward Euler method. **c** The compressed graph representation and the Backward Euler method. **d** The compressed graph representation and the Backward Euler method while employing ideal positions attraction forces

**Fig. 10:**
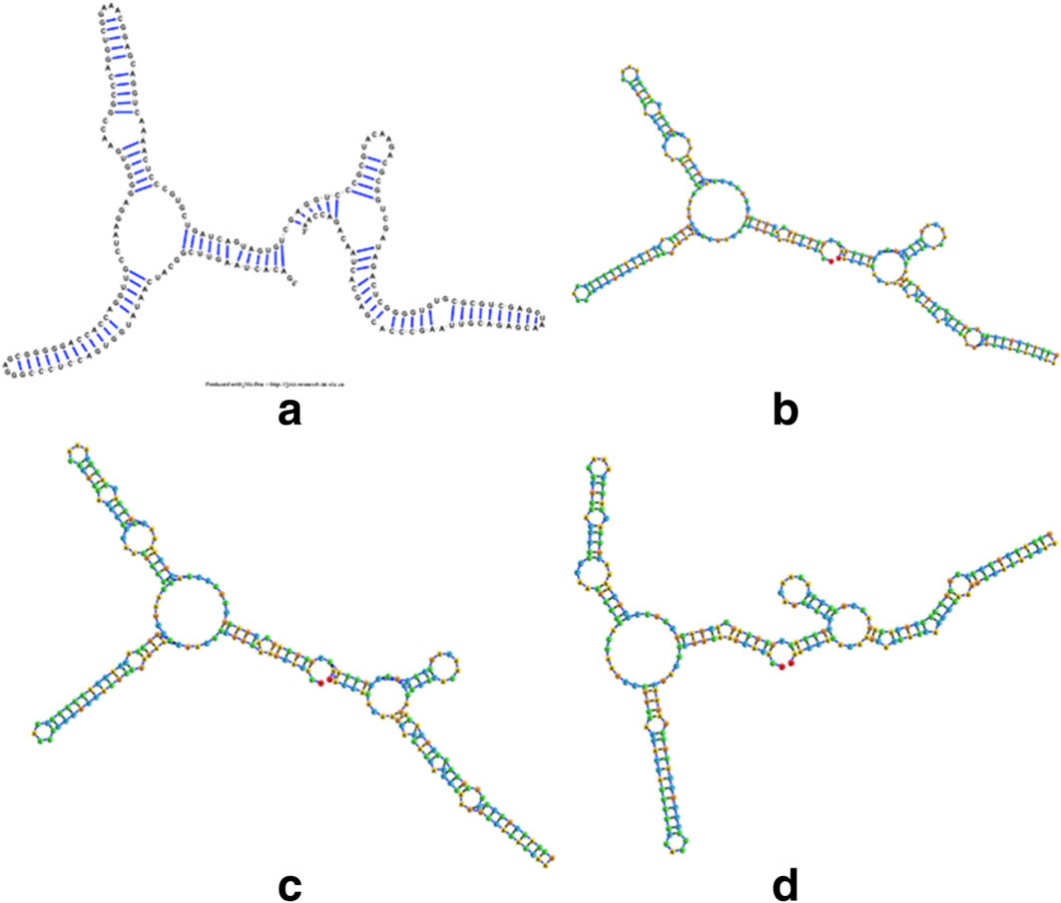
The visualization result obtained for the 217 nt RNA (RNA STRAND ID PDB_01076) utilizing: **a** The detailed graph representation (employing the Forward Euler method. **b** The compressed graph representation and the Forward Euler method. **c** The compressed graph representation and the Backward Euler method. **d** The compressed graph representation and the Backward Euler method while employing ideal positions attraction forces

**Fig. 11:**
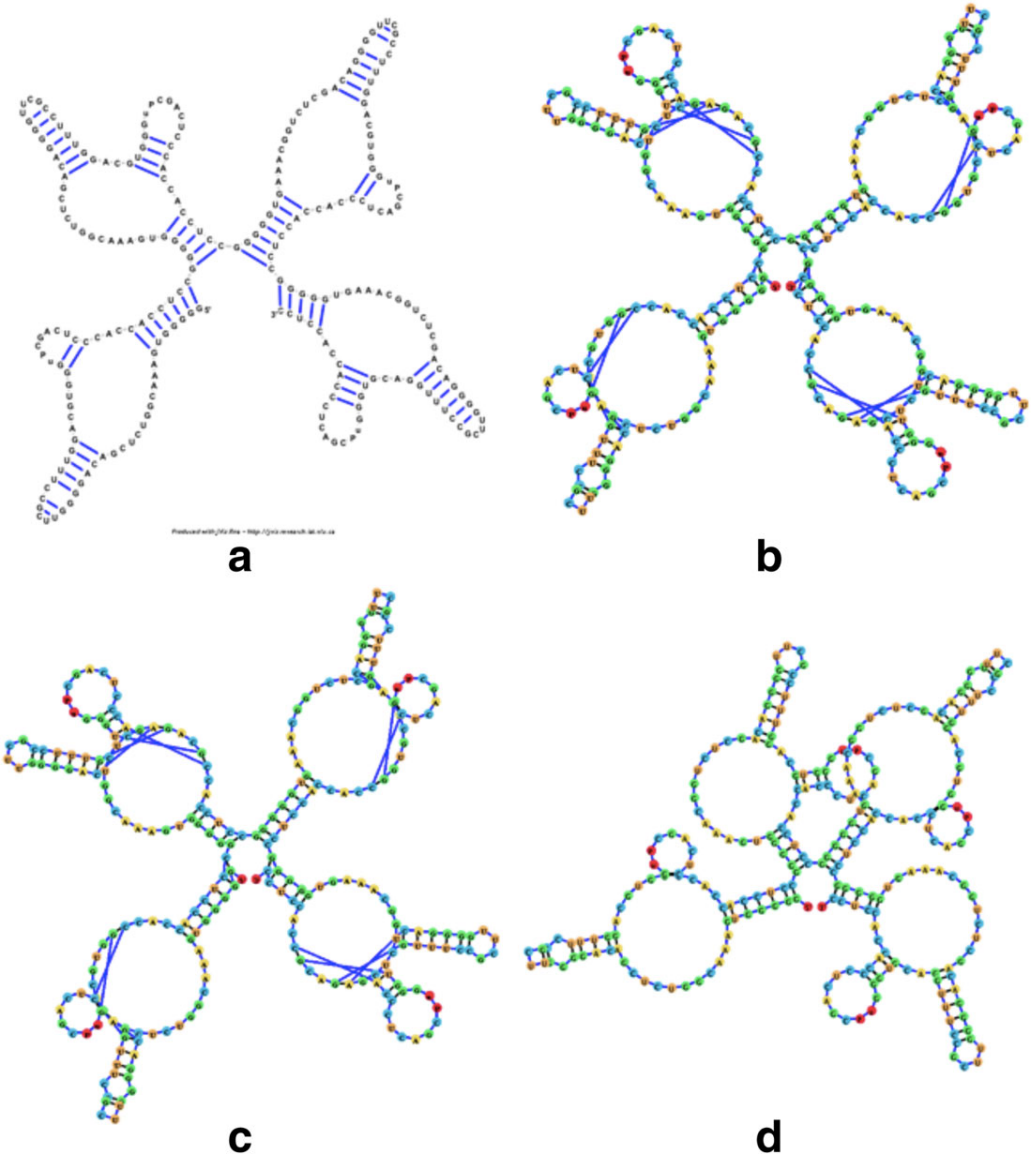
The visualization result obtained for the 248 nt RNA (RNA STRAND ID PDB_00985) utilizing: **a** The detailed graph representation (employing the Forward Euler method). **b** The compressed graph representation and the Forward Euler method. **c** The compressed graph representation and the Backward Euler method. **d** The compressed graph representation and the Backward Euler method while employing ideal positions attraction forces

**Fig. 12:**
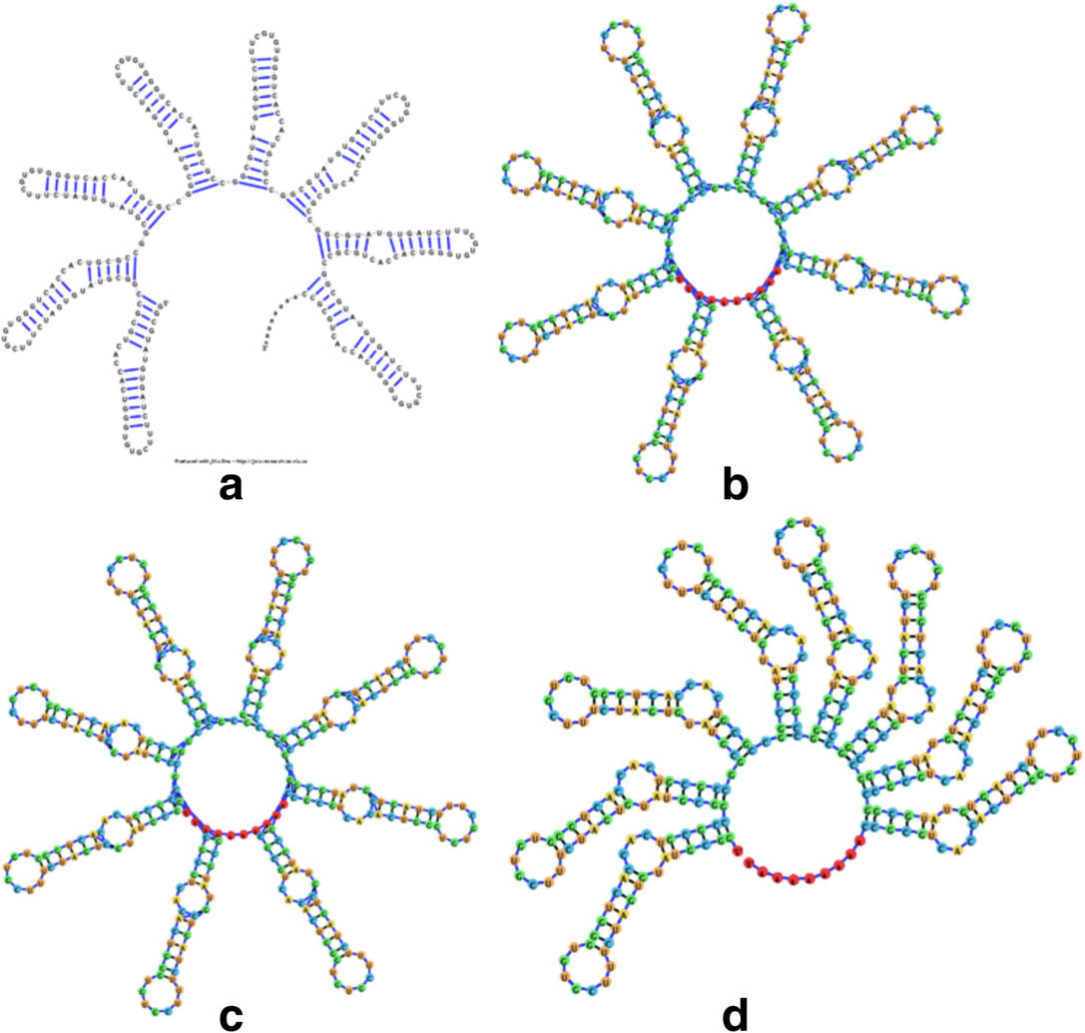
The visualization result obtained for the 304 nt RNA (RNA STRAND ID PDB_00528) utilizing: **a** The detailed graph representation (employing the Forward Euler method). **b** The compressed graph representation and the Forward Euler method. **c** The compressed graph representation and the Backward Euler method. **d** The compressed graph representation and the Backward Euler method while employing ideal positions attraction forces

**Fig. 13:**
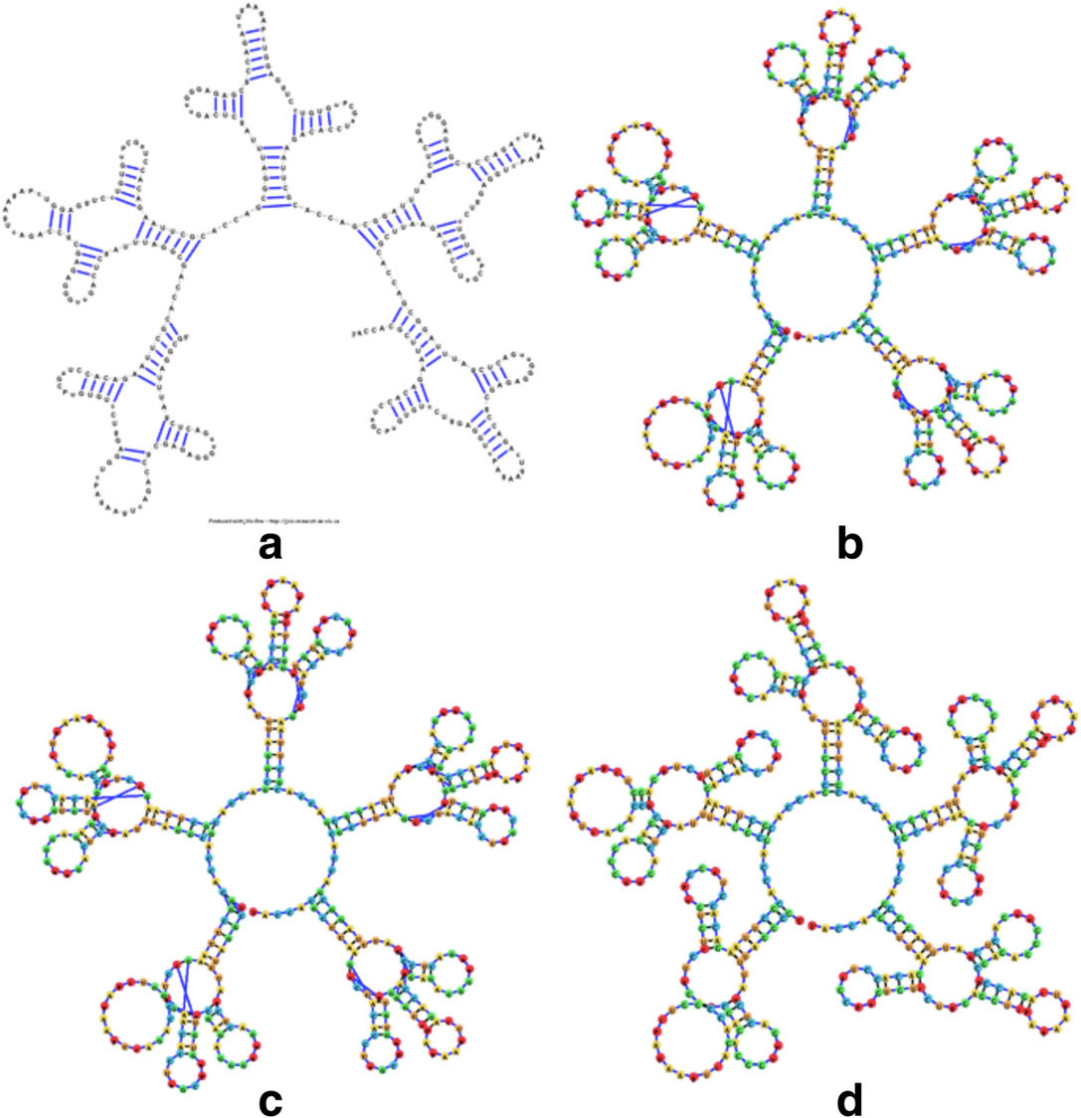
The visualization result obtained for the 380 nt RNA (RNA STRAND ID PDB_00398) utilizing: **a** The detailed graph representation (employing the Forward Euler method). **b** The compressed graph representation and the Forward Euler method. **c** The compressed graph representation and the Backward Euler method. **d** The compressed graph representation and the Backward Euler method while employing ideal positions attraction forces

In order to get a full appreciation of the advantages of the different methods explored in this work, as well as potential future improvements, it is necessary to look at both the run-times reported in Figs. 6 and 7, as well as at the resulting visualizations each method produced. Figures 8, 9, 10, 11, 12 and 13 demonstrate the visualizations produced when employing the four different methods explored in this paper (all Euler implementation of the compressed graph as well as their detailed graph counter-part).

Comparing the images produced by the compressed and detailed graphs reveals additional differences between the methods. It is immediately evident that the figures produced by employing the compressed graph adhere more strictly to RNA visualization conventions; namely, the circular loops, and the constant distance between base pairs. However, at the same time, the detailed graph representation demonstrates some advantages over the compressed graph images. First, there are no cases of stems that intersect, which contributes to less user intervention being required to “untangle” the structure. The compressed graph representations, on the other hand, occasionally have stems that intersect and would require the user to explore the structure to resolve such conflicts. Though this drawback is addressed to a large extent by substituting the ideal positions as the attraction points for the vertexes, the need to manually untangle the structure may persist in certain cases.

**Fig. 14:**
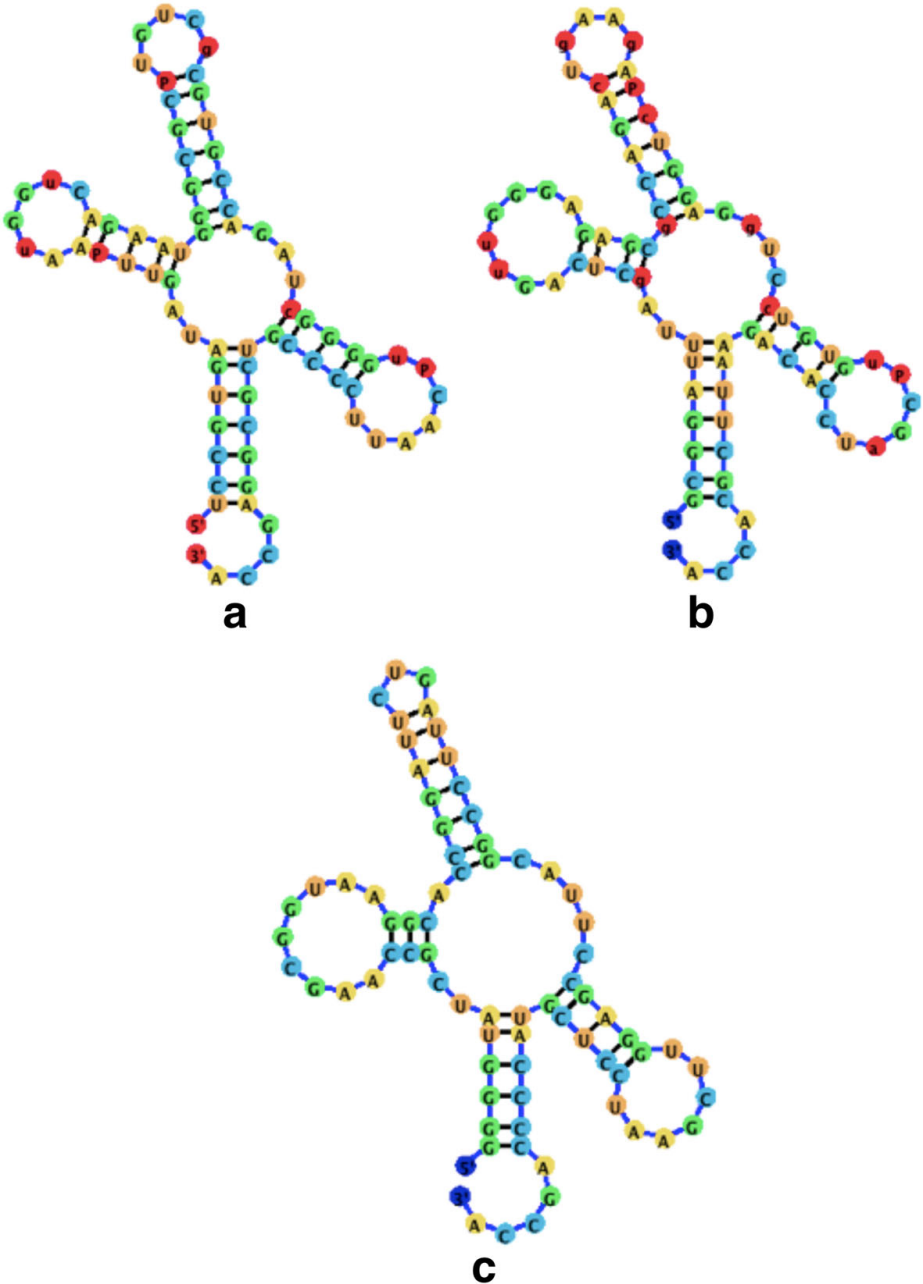
A visualization comparison of tRNA molecules employing the Backward Euler method and the ideal positions attraction forces. **a** The visualization for a Yeast tRNA utilizing jViz.RNA (RNA STRAND ID: NDB_00051, PDB ID: 1VTQ). **b** The visualization for a Yeast tRNA utilizing jViz.RNA (RNA STRAND ID: PDB_00045, PDB ID: 1EHZ). **c** The visualization for an E.coli tRNA utilizing jViz.RNA (RNA STRAND ID: PDB_00426, NDB ID: 1GTS)

Figure 14 demonstrates the layout algorithm as it is applied to a few instances of related RNAs. All three RNA molecules shown in Fig. 14 are tRNA molecules for different amino acids. The utilization of the ideal layout algorithm allows the related RNA molecules to be laid out in a similar conformation. Though different users may wish to align RNA structures differently, related RNA molecules should be drawn in a similar fashion to easily highlight homologous structural regions, which may share functional roles (such as the anticodons located on the tRNA middle stem, and the binding site for the amino acid located at the 3’ end).

## Discussion

Two major objectives have been set out for this work: The first was to improve jViz.RNA’s visualization through a new representation, the second was to design an enhanced automatic layout algorithm in light of this new representation and to improve its run-time performance. Both objectives have been achieved through our employment of the compressed graph representation and the Backward Euler method.

### Improving jViz.RNA’s visualization

Comparing the detailed graphs and compressed graph visualization demonstrated that employing the compressed graphs produces visualizations more consistent with current RNA visualization methods, and does so at a fraction of the time. However, for large molecules that gain in time may be offset by the time required by the user to examine and untangle the RNA structure in the case of intersecting stems. This can be addressed by the modification of the attraction force to act as both an attraction and rotation force. However, the existence of some overlap may still be present for certain structures (such as those seen in Fig. 11 d). Future work to address this limitation would focus on configuring the system to find an equilibrium between the correct positioning of stems and repulsion between the various RNA components to prevent them from overlaying each other.

### Extending the automatic layout algorithm

Comparing the two methods of calculating movement for the RNA components of the compressed graph revealed that employing the Backward Euler method produces more stable simulations of the vertexes’ movement, and does so in less time than the Forward Euler method (Figs. 6 and 7). Though it is evident the connectivity of the structure determines the degree of run-time improvement, it is evident that all structures we have tested stabilize faster using the Backward Euler method. Given that the Backward Euler implementation is much more stable than its Forward counter-part, it is the Backward Euler method that becomes the more desirable choice.

## Conclusion

In this article we described advances made in the representation and layout algorithms for dynamic RNA secondary structure visualization. We reviewed existing tools and algorithms and showed that only few allow for dynamic visualization. One such tool is jViz.RNA. We discussed its shortcomings in terms of layout, stability and run-time performance and proposed several improvements based on a compressed graph representation and advanced numerical integration methods. We presented two graph based representations for RNA visualization, as well as two methods to create dynamic RNA structures that lay themselves out automatically and respond to user interaction.

The utilization of compressed graphs as a model of the RNA structure, the experiments shown here profiling their performance, and examining the underlying physics, demonstrate a substantial improvement to the original representation and layout algorithms employed in jViz.RNA. The work on layout conventions was greatly influenced by feedback from life science collaborators. The new algorithms have increased stability for automatic layout, reduced overlap of structural elements that decreases the need for user intervention, and increased run time performance to allow for the handling of larger RNA structures. Having examined the basic properties of compressed graph behaviour, we can verify it is a better tool to produce dynamic and responsive RNA models than its detailed counter-part. We have also discovered a few important areas of improvement such as stem orientation relative to loops, and prevention of structural element intersection, and provided improvements to the layout algorithm to handle these issues. By comparing the Forward and Backward Euler methods we demonstrated the superiority of the Backward Euler method in its ability to support larger time steps without losing stability, and consequently, allowing for faster simulations of the RNA compressed graph.

Our future work will focus on incorporating pseudoknot representation into the compressed graph model, to allow for the visualization of pseudoknotted structures. Additionally, constructing and modifying RNA structures will also be explored.

Overall, this manuscript has explored the use of compressed graphs to improve the layout of RNA secondary structures, as well as the best physics based simulation method for such an implementation. The results presented demonstrate that both the compressed graph representation, as well as the Backward Euler integrator, greatly enhance the run-time performance and usability.

We anticipate these findings to benefit other researchers in RNA structure visualization, or more generally, biological structure visualization, as the underlying ideas are transferrable. We also provide a tool that is platform independent, easy to use, and can quickly render publication quality structure images. We would anticipate that many researchers in RNA structure will find this tool useful and that it will find wide acceptance. We believe the technical details discussed in this manuscript will impact how other visualization researchers think about dynamic structure visualization and we see the impact of this work in both providing a useful software tool as well as presenting a methodology for other visualization tools to adopt.

## Endnote

^1^ The original IDs of the structures were obtained from the Comparative RNA Website (CRW) [35], the Protein Data Bank (PDB) [36], the Signal Recognition Particle Database (SRPDB) [37], and the RNA Families Database (Rfam) [38].

## Additional file

Additional file 1 The file jViz3.0_Complete is a zip file containing a Java executable file (jViz3.0.jar), a User Manual in PDF format (jViz3.0 User Manual), and a subfolder containing RNA secondary structure files in.ct format for the RNA structures visualized in this manuscript (RNA_Structures). (ZIP 843 kb)

## Abbreviations

CRW: Comparative RNA Website
DNA: Deoxyribo-nucleic Acid
nt: nucleotide
PDB: Protein Data Bank
Rfam: RNA Families Database
RNA: Ribo-nucleic Acid
rRNA: Ribosomal RNA
SRPDB: Signal Recognition Particle Database
tRNA: Transfer RNA
UTR: Untranslated Region
