# Supplementary material for: Numerical integration methods and layout improvements in the context of dynamic RNA visualization
Source: BMC Bioinformatics. 2017 May 30;18:282. doi: 10.1186/s12859-017-1682-0 (PMC5450055; doi:10.1186/s12859-017-1682-0)
Supplement: Additional file 1 — The file jViz3.0_Complete is a zip file containing a Java executable file (jViz3.0.jar), a User Manual in PDF format (jViz3.0 User Manual), and a subfolder containing RNA secondary structure files in.ct format for the RNA structures visualized in this manuscript (RNA_Structures). (ZIP 843 kb) [file 12859_2017_1682_MOESM1_ESM.zip › jViz3.0/jViz3.0 Quickstart Manual.pdf]

Simon Fraser University
School of Computing Science

jViz.RNA 3.0 Quick Start Manual

BORIS SHABASH

KAY C. WIESE

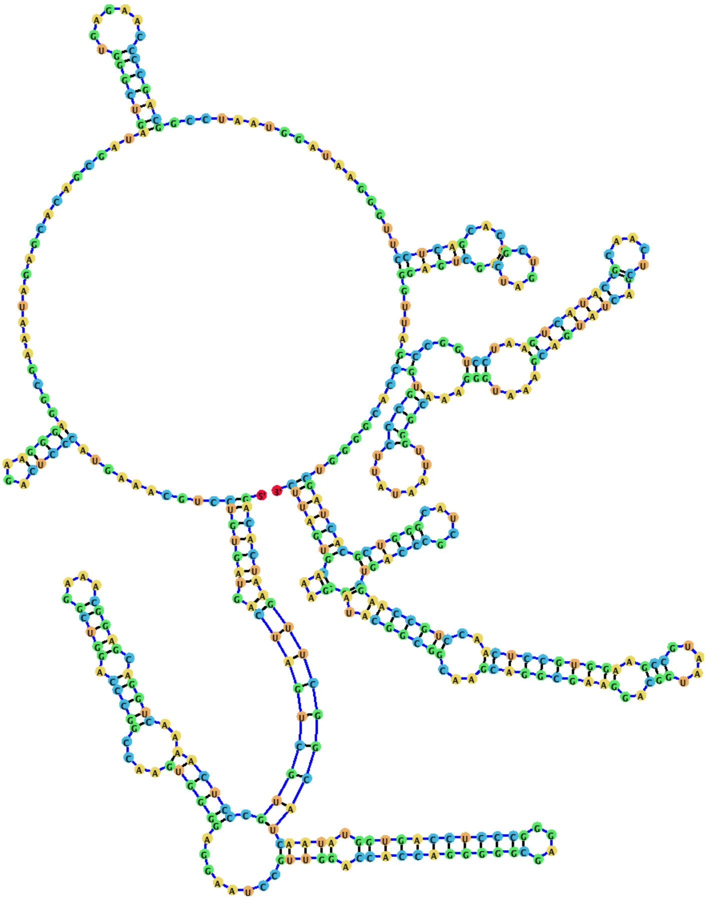

Loading RNA Secondary Structures

Running the software

The jViz.RNA complete package can be downloaded from `https://jviz.cs.sfu.ca/download/download.html`. jViz.RNA requires no installation since it is a java `.jar` executable. In order to run the software, simply unzip/unpack the contents of the folder `jViz.RNA 3.0`. The folder should contain the `jViz.jar` file, and a folder of RNA secondary structures `RNA Structures`.

Running under Mac OS X and Windows:

Double-clicking the `.jar` executable launches jViz.RNA 3.0 using the default `Java` environment

Running under Ubuntu:

In order to run jViz.RNA 3.0 under Ubuntu, simply right-click on the `.jar` executable and select `Open With -> Java Runtime Environment`

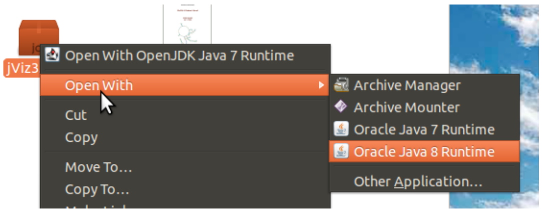

Running from the command terminal

In Windows, Mac OS X, and Ubuntu, jViz.RNA 3.0 can be launched by navigating to the folder containing the `.jar` file, and calling the command:

```
java -jar jViz3.0.jar
```

Visualizing the RNA structures

To load an RNA structure simply go to `Load File -> Load File For Structure Visualization` and select the file from the `RNA Structures` folder.

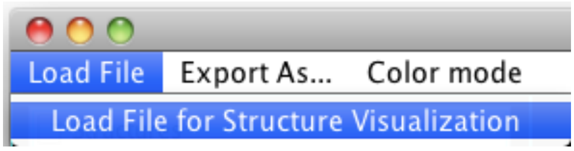

Testing the software

We have tested the software on a wide variety of systems to confirm its stability and functionality. The tests included running jViz.RNA 3.0 on Windows 7, Mac OS X 10.6.8 and 10.11.6, and Ubuntu version 14.04.5. The software was tested under different Java versions entailing 1.7.0_77-b03, 1.7.0_80-b15, 1.8.0_91-b14, 1.8.0_102-b14 and 1.8.0_-112-b15. In total, 22 machines were tested with no faults detected.

On one machine, however, we did notice an unusual behaviour where the Load file dialogue window **needed to be resized** before it renders its contents. We subsequently tested jViz.RNA 3.0 on a machine that is identical in hardware, operating system, and Java Version to the machine that produced the unusual behavior and were unable to reproduce the error. We believe that it is a machine related issue. Given our test protocol above we are very confident in the stability of jViz.RNA 3.0. However, in the unlikely event that the Load File window does not render as expected, **simply resizing it promotes proper rendering.**

Manipulating the RNA Secondary Structure

Automatic Layout

jViz.RNA uses a force based system to calculate an automatic layout for the RNA molecule. The automatic layout structure is very quick, but can still take several seconds for very large structures. If you wish to stop the automatic layout process for any reason, push the `STOP FORCE CALCULATIONS` button at the bottom of the main panel.

Manual Layout

The loops and base-pairs can be manipulated by selecting them and dragging them to their desired positions. Once a base-pair or loop has been selected and/or moved, it is "locked" into its position and will not move (locked loops and base-pairs have a red hue around them). **Double-clicking** the base-pair or loop will **unlock** it, and return it the position determined by the automatic layout algorithm. **Single-clicking** the loop/base-pair while it is unlocked **locks** it again.

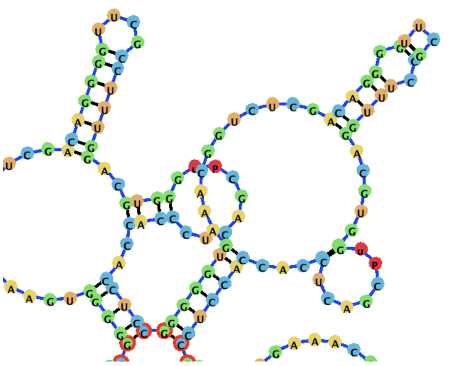

(a) *Two nodes are intersecting in the final RNA layout. They need to be separated by the user*

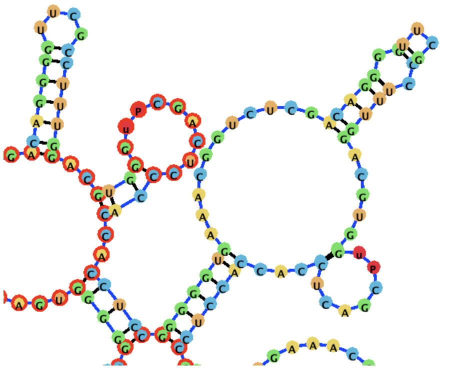

(b) *The nodes have been moved and "locked" into place (as evident by the red hue around them)*

Exporting the RNA Secondary Structure

Exporting as an image

Currently, jViz.RNA 3.0 allows for `PNG` file export. To export your structure as a `PNG` file, go to `Export as... -> Export as Image File -> Export as PNG Image` and select a name as well as a save destination.

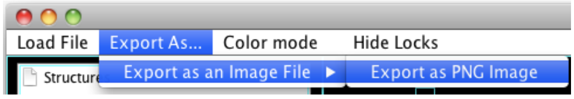

Removing the locked red hue

When preparing final images of the RNA molecules, it can be inconvenient for the image to contain the red hue which denotes "locked" parts of the molecule. To remove the red hue, click on `Hide Locks` at the right-most side of the top menu. To bring the red hue back, click on `Show Locks` at the same place. Hiding the red hue DOES NOT remove the locks, it simply hides the lock indicator. To "unlock" the loops or base-pairs, simply double click them.

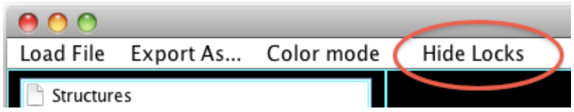

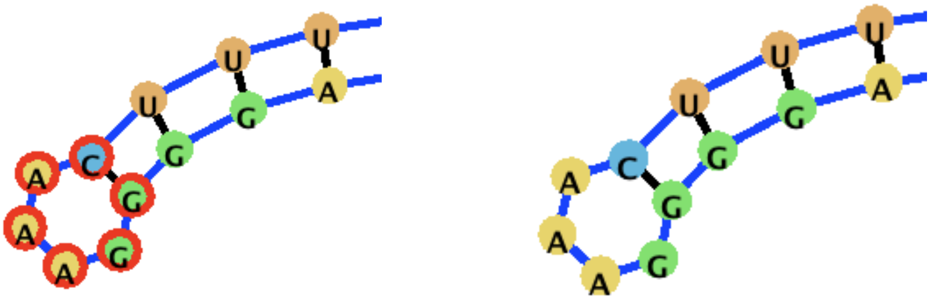

(a) *The loop has been locked into place by the user, and has a red hue to indicate it is locked*

(b) *The loop is still locked. However the red hue has been removed for visualization purposes.*

Colour-blind mode

By default, the colour scheme of jViz.RNA 3.0 is `Regular colour mode`. To change to a colour-blind friendly representation of the nucleotides, simply go to `Colour mode -> Colour-blind mode`. The nucleotides can also have their colour removed by selecting `Colour mode -> No Colour mode`.

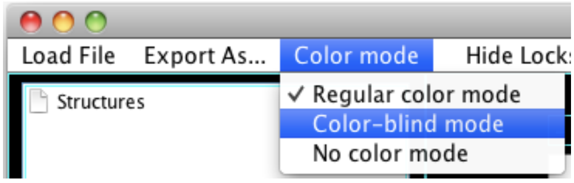

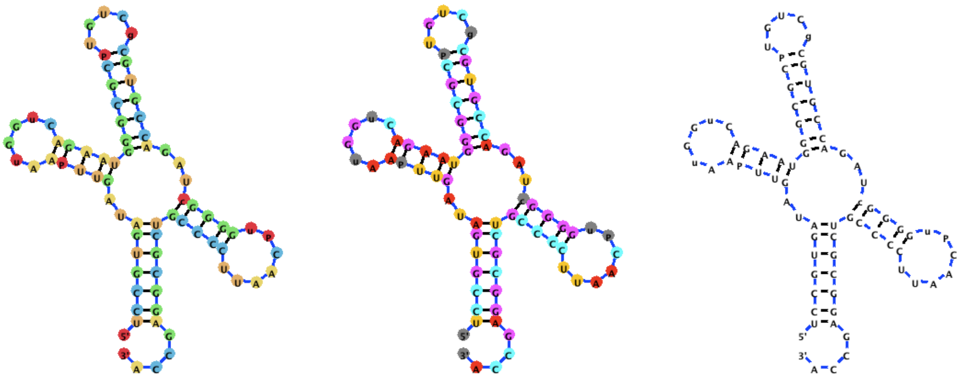

(a) *A sample RNA structure viewed in regular mode*

(b) *The sample RNA structure viewed in colour blind mode*

(c) *The sample RNA structure viewed in no colour mode*
